# Overexpression of *lncRNA77580* Regulates Drought and Salinity Stress Responses in Soybean

**DOI:** 10.3390/plants12010181

**Published:** 2023-01-01

**Authors:** Xiangqian Chen, Xuemin Jiang, Fengjuan Niu, Xianjun Sun, Zheng Hu, Fei Gao, Hui Zhang, Qiyan Jiang

**Affiliations:** 1 Institute of Crop Science, Chinese Academy of Agricultural Sciences, Beijing 100081, China; 2 College of Life and Environmental Sciences, Minzu University of China, Beijing 100081, China

**Keywords:** soybean, drought stress, salt stress, long non-coding RNA

## Abstract

Emerging evidence indicates that long non-coding RNAs (lncRNAs) play important roles in diverse biological processes. However, the biological functions of most plant lncRNAs are still unknown. We previously discovered a soybean abiotic-stress-related lncRNA, *lncRNA77580*, and cloned the entire full-length sequence. Here, in order to fully identify the function of *lncRNA77580* in soybean stress response, we created transgenic soybean lines overexpressing *lncRNA77580*. Compared with the wild type, overexpression of *lncRNA77580* enhances the drought tolerance of soybean. However, the transgenic plants exhibit increased sensitivity to high salinity at the seedling stage. We found that *lncRNA77580* modulates the transcription of different gene sets during salt and drought stress response. Under water deficit at the reproductive stage, *lncRNA77580* overexpression increases the seed yield by increasing the seed number per plant. These results provide insight into the role of *lncRNA77580* in soybean stress response.

## 1. Introduction

Soybean (*Glycine max* (L.) Merrill.) is a highly nutritious leguminous crop that is widely used as a food source for humans and livestock due to its rich contents of proteins and minerals [1,2]. Furthermore, soybean is also a major oilseed crop, representing around 59% of the overall world oilseed production [3]. However, the growth and yield of soybean, similarly to other crops, are largely affected by abiotic stresses, including drought and salinity stresses. As an example, drought alone can cause up to a 40% yield loss of soybean globally [4]. This also severely limits the space for soybean growth and limits the potential of saline–alkali and arid land to be used as back-up farmland. To solve this problem, it is very important to explore and utilize highly effective stress-resistance genes in soybean.

Long non-coding RNAs (lncRNAs) are RNAs generally longer than 200 nucleotides (nt) that lack protein-coding capability. Emerging evidence indicates that lncRNAs play key roles in gene regulation, development, and environmental responses [5,6,7,8]. Animal lncRNAs have been extensively studied and proven to be functional in different essential biological processes. Large batches of plant lncRNAs have been identified in recent years [9,10,11,12,13], taking advantage of next-generation sequencing technologies. In soybean, 6018 lincRNAs have been identified from transcriptomic data [14]. In our earlier study, we found that more than 75% of discovered lncRNAs in soybean roots were activated or upregulated by continuous salt stress [15]. From two genotypes of soybean grown under different levels of P, 4166 novel lncRNAs, including 525 differentially expressed (DE) lncRNAs, were identified [16].

The functional roles of plant lncRNAs remain largely unknown, and only a few plant lncRNAs are well understood. For example, the rice-specific lncRNA *LDMAR* has been identified as a key gene in controlling photoperiod-sensitive male sterility [17]. Overexpression of the lncRNA *LAIR* in rice increases grain yield and regulates the expression of genes in a neighboring cluster, which is known as cis-regulation [18]. Two lncRNAs, *COOLAIR* and *COLDAIR*, finely regulate flowering in *Arabidopsis* [19,20]. In addition, a few lncRNAs were found to play an important role in abiotic stress response through different regulation mechanisms. In *Arabidopsis*, lncRNA *SVALKA* governs plant cold acclimation by the *SVALKA–asCBF1* cascade mechanism, tightly controlling the level and timing of *CBF1* expression, which can be exploited to maximize freezing tolerance with mitigated fitness costs [21]. At the post-transcriptional level, some lncRNAs can interfere with microRNAs (miRNAs), neutralizing their silencing role and thus upregulating the transcript level in response to cold stress [22], heat stress [23], etc. A nucleus-localized *Arabidopsis* lncRNA, *DRIR*, enhances drought and salt stress tolerance by modulating the expression of a series of genes involved in the stress response [24]. These studies reveal the key roles of plant lncRNAs in appropriately acclimating to challenging environmental conditions. However, little is known about the functions of lncRNAs in soybean.

In our previous study, we identified a salt-stress-related lncRNA in soybean, *lncRNA77580* [15], which was identified as an intergenic lncRNA located on chromosome 20 between the genes *Glyma.20G225700* and *Glyma.20G225800*. We cloned the full length of *lncRNA77580* with a 4851 bp DNA fragment and found through overexpression and large DNA fragment deletion in soybean that *lncRNA77580* regulates the expression of neighboring genes [25]. In this study, we further investigate the function of *lncRNA77580* in transgenic soybean in response to drought and salt stress to support its proposal as a candidate gene for soybean stress tolerance gene mining.

## 2. Results

### 2.1. Creation of Transgenic Soybean Overexpressing lncRNA77580

To evaluate the contribution of *lncRNA77580* in salt and drought stress, we engineered soybean plants to constitutively express *lncRNA77580* under a 35S promoter (Figure S1). We selected three independent stable homozygous lines (OE-1, OE-2, and OE-3) for functional analysis. The expression of *lncRNA77580* in the three transgenic lines was validated through qPCR (Figure S1). The results of qPCR analysis show that *lncRNA77580* transcript levels are significantly higher in *lncRNA77580*-OE soybean than in the corresponding WT (wild type) lines under normal conditions (Figure S1).

### 2.2. Transgenic Soybean with lncRNA77580 Overexpression Exhibits Increased Sensitivity to High Salinity

**Figure 1:**
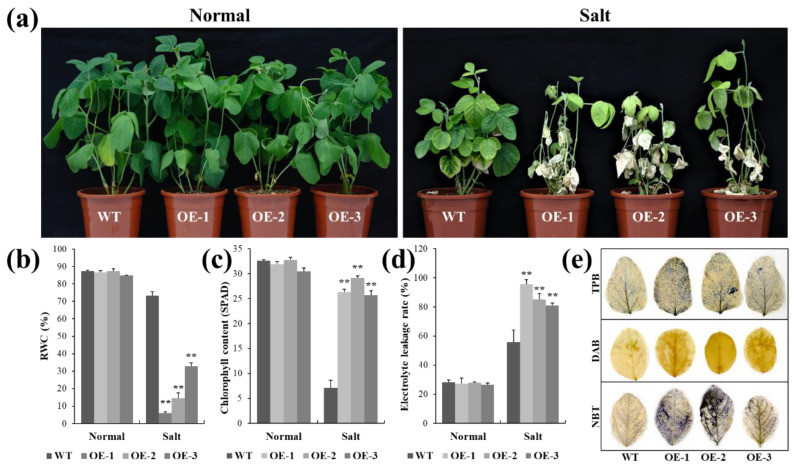
Phenotypic and physiological analyses of the WT plants and *lncRNA77580*-OE transgenic soybean under salt stress. (**a**) Phenotypes of overall plants and (**b**) relative water content, (**c**) chlorophyll content, (**d**) electrolyte leakage rate, and (**e**) trypan blue, DAB, and NBT staining of the leaves of the WT plants and *lncRNA77580*-OE transgenic soybean grown under salt treatment or normal control conditions for 35 days. Data are shown as the mean ± standard deviation (n = 3). Significant differences based on ANOVA were set at *p* < 0.01 (**).

To further verify the biological function of *lncRNA77580* in soybean, a *lncRNA77580* overexpression vector was transferred into the soybean cultivar Tian Long via *Agrobacterium tumefaciens*-mediated transformation, and the phenotypes of seedlings under salt stress were evaluated. Under normal conditions, the WT and lncRNA77580-OE soybean seedlings showed similar growth phenotypes. In the experimental group, soybean seedlings were grown in vermiculite and continuously treated with 100 mmol/L NaCl solution starting from seed germination. At 35 days of salt treatment, the leaves of the wild-type and transgenic soybean had turned yellow, and most leaves of the transgenic plants were completely dry, as obvious from their phenotype (Figure 1a). Unlike the OE lines, most of the WT plants had evidently retained chlorophyll in their leaves, indicating continued support of essential life processes.

Two physiological indices, i.e., chlorophyll content as an indicator of photosynthetic capacity [26] and relative water content (RWC) as an indicator of plant water status, were measured to quantify the effects of salt and drought on the plant development [27]. The changes in the RWC (Figure 1b) and chlorophyll content (Figure 1c) of plant leaves in response to drought and salt were consistent with the observation of leaf phenotypes. Specifically, the leaves of *lncRNA77580* OE lines showed a lower RWC and chlorophyll content than did those of the WT plants.

In order to visually show the degree of damage in soybean WT and OE plant leaves, we used trypan blue, DAB, and NBT staining to measure cell viability in soybean leaves under salt stress. The WT plant leaves were significantly less stained by all three staining methods than the OE plant leaves, suggesting that the leaves of *lncRNA77580* OE plants suffered more damage under salt treatments than the WT plants (Figure 1e). The electrolyte leakage rate of the leaves was significantly higher in OE plants than in WT (Figure 1d). These results indicate an evident growth disadvantage for the *lncRNA77580* OE lines compared with the WT plants under salt stress.

### 2.3. lncRNA77580 Positively Regulates Soybean Tolerance to Drought Stress

**Figure 2:**
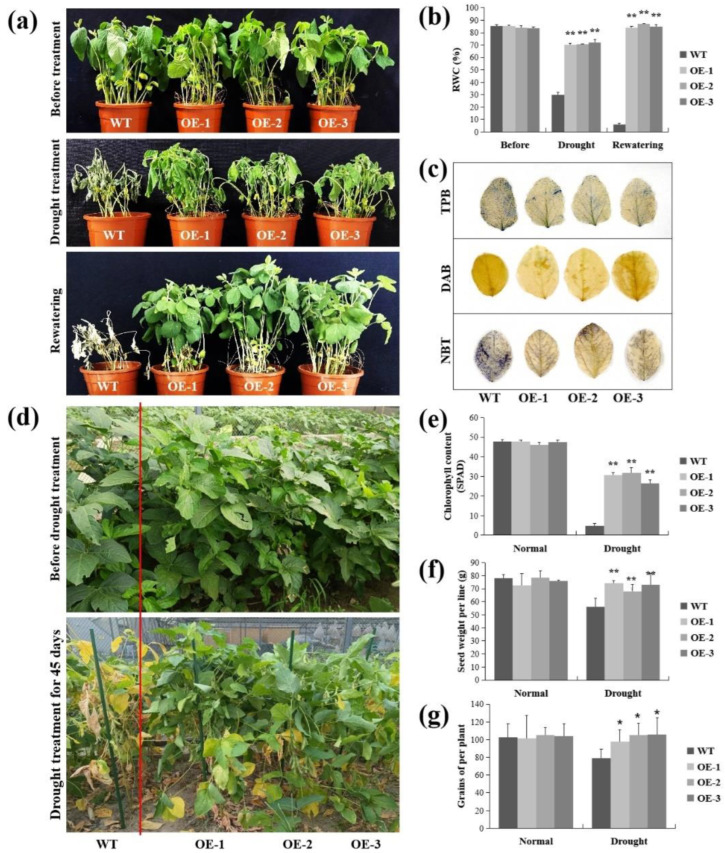
Phenotypic and physiological analyses of WT plants and *lncRNA77580*-OE transgenic soybean under drought stress in growth chambers (**a**–**c**) vs. the field (**d**–**g**). (**a**) Phenotypes of overall plants and (**b**) relative water content and (**c**) trypan blue, DAB, and NBT staining of the leaves of the WT plants and *lncRNA77580*-OE transgenic soybean grown under drought treatment or well-watered conditions. (**d**) Phenotypes of overall plants and (**e**) chlorophyll content, (**f**) seed weight per line, and (**g**) seed number per plant of the WT plants and *lncRNA77580*-OE transgenic soybean grown under drought treatment or in a well-watered field. Data are shown as the mean ± standard deviation (*n* = 3). Significant differences based on ANOVA were set at *p* < 0.05 (*) and *p* < 0.01 (**).

The *lncRNA77580* overexpression soybean was similarly treated with drought stress. At the seedling stage, the WT and *lncRNA77580*-OE soybean showed similar growth phenotypes under well-watered conditions (Figure 2a). At the fifth day of drought treatment, the three *lncRNA77580*-OE lines exhibited more robust growth than the WT plants, as indicated by significantly less leaf wilting, curling, and chlorosis (Figure 2a). After rewatering for five days, the control plants were unable to recover and eventually died. By contrast, the majority of *lncRNA77580*-OE transgenic soybean seedlings appeared to be healthy after rewatering (Figure 2a). Consistent with these results, leaf RWC was significantly higher in the *lncRNA77580*-OE lines than in WT plants (Figure 2b), and the transgenic plants suffered less damage than the WT plants under drought treatments, as indicated by less intense staining with trypan blue, DAB, and NBT (Figure 2c). Thus, constitutive overexpression of *lncRNA77580* positively regulates soybean tolerance to drought stress. Overexpression of *lncRNA77580* in soybean has opposite effects on responses when comparing between drought and salt stress.

In addition, we planted WT and *lncRNA77580*-OE soybeans in a field with an automatic control canopy. The control group was well-watered, and the WT and OE soybeans in the experimental group were not watered after flowering. At 45 days of drought treatment, most leaves of the WT soybean had turned yellow or completely dried, while most of the leaves in the three *lncRNA77580*-OE lines remained green (Figure 2d,e). After the soybeans were ripe and harvested, we investigated some agronomic traits. The results show a significantly lower reduction due to drought stress in the seed yield and seed number per plant (Figure 2f,g), demonstrating once again that transgenic soybean lines have higher drought resistance ability than the WT.

### 2.4. lncRNA77580 Modulates the Expression of a Series of Genes Involved in the Stress Response

The *lncRNA77580*-OE soybean showed a salt-sensitive and drought-tolerant phenotype compared with WT. It was hypothesized that this may result from differences in how the expression of some genes associated with stress responses may be changed. Therefore, RNA-seq was conducted to identify which genes are differentially expressed (referred to as DEGs) between the *lncRNA77580*-OE and WT plants under normal (WT-N, OE-N), salt (WT-S, OE-S), and drought (WT-D, OE-D) stress.

**Figure 3:**
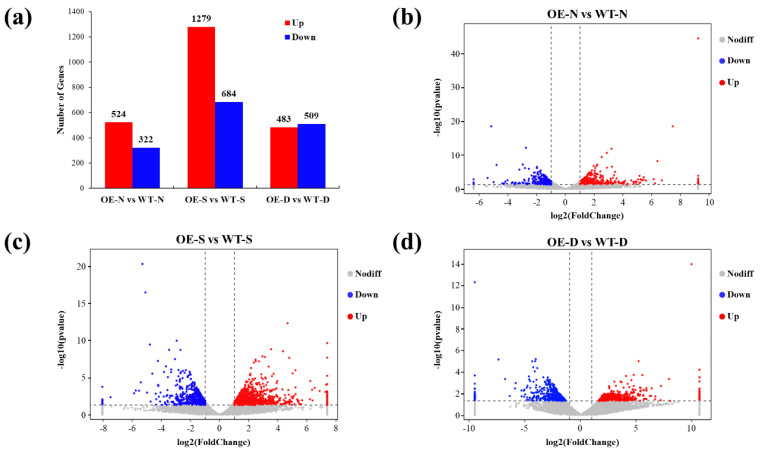
DEGs of soybean seedlings overexpressing *lncRNA77580* under normal, salt, and drought conditions. (**a**) Numbers of up- and downregulated DEGs in *lncRNA77580*-overexpressing soybean (OE) under normal (N), salt (S), and drought (D) conditions. (**b**–**d**) Volcano diagrams of DEGs in *lncRNA77580*-overexpression soybean under normal, salt, and drought conditions compared with wild-type (WT) soybean. Blue spots represent DEGs whose expression was less than half that of the level in WT. Red spots represent DEGs whose expression was more than double that of the level in WT.

Overexpression of *lncRNA77580* in soybean alters the transcript profile under normal and stress conditions (Figure 3). There were 524 upregulated DEGs and 322 downregulated DEGs in OE-N compared with WT-N, 1279 upregulated DEGs and 684 downregulated DEGs in OE-S compared with WT-S, and 483 upregulated DEGs and 509 downregulated DEGs in OE-D compared with WT-D (Figure 3). These data suggest that *lncRNA77580* modulates the transcription of genes during salt and drought stress responses. These genes also include many transcription factors. Members from all of the major transcription factor (TF) families (such as ERF/AP2, ZFP, MYB, bHLH, NAC, bZIP, and WRKY) were found to be involved in the regulation of *lncRNA77580* under salt and drought stress based on their altered pattern of expression (Figure S3).

**Figure 4:**
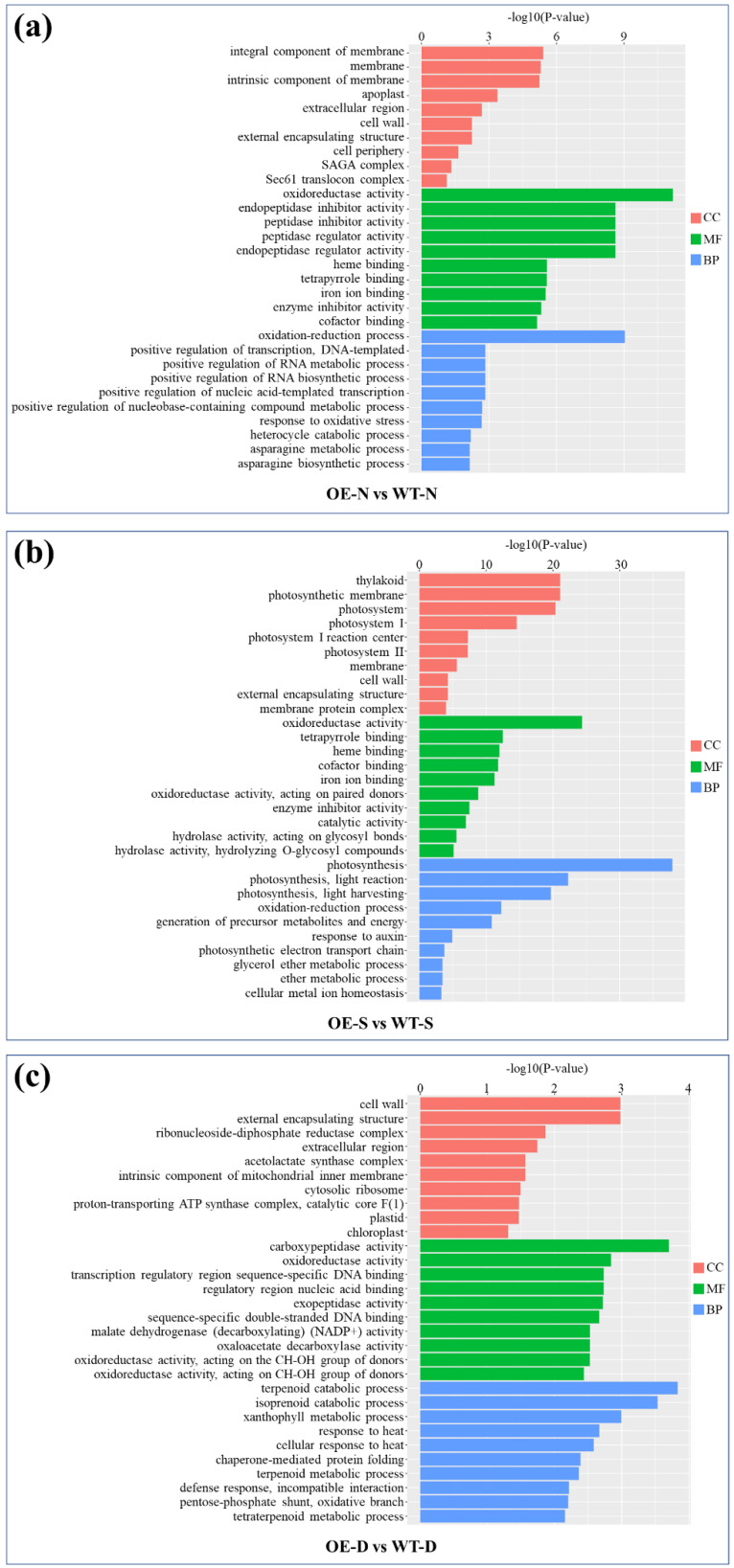
Gene Ontology analysis of the differentially expressed genes and their associated terms in molecular functions (MF), cellular components (CC), and biological processes (BP). (**a**–**c**) GO enrichment analysis of DEGs in *lncRNA77580*-OE soybean compared with WT under normal (N), salt (S), and drought (D) conditions.

Gene Ontology (GO) analysis of the DEGs revealed potential functions of *lncRNA77580* according to three terms: biological process, molecular function, and cellular component (Figure 4). Under the three different conditions, normal, salt, and drought, few of the represented terms are the same. For example, cell wall (GO: 0005618) for cellular component and oxidoreductase activity (GO: 0016491) for molecular function are common and represent the exception. Under salt stress, *lncRNA77580* overexpression had a greater impact on the expression of genes related to photosynthesis, and most are associated with terms for locations in the thylakoid, photosystem, and membrane (GO: 0009579, GO: 0009521, and GO: 0016020). Meanwhile, under drought stress, for cellular component, the major category is cell wall (GO: 0005618), and the genes most significantly enriched among all terms are associated with terpenoid catabolic process (GO: 0016107). These data suggest that *lncRNA77580* modulates the transcription of different gene sets during salt and drought stress responses.

## 3. Discussion

Although not as well studied as in animals, it is known that lncRNAs, in plants, can regulate gene silencing, flowering time, reproduction, stress responses, organogenesis in roots, and photomorphogenesis in seedlings [11,28,29,30,31,32]. However, the overwhelming majority of plant lncRNAs with clear functions were studied in *Arabidopsis*, and our understanding of the lncRNAs in crop species remains limited [18]. In this study, we discovered that overexpression of a soybean lncRNA, *lncRNA77580*, can result in an obvious increase in soybean drought tolerance. However, the transgenic plants exhibited increased sensitivity to high salinity. Similar results have been obtained using transgenic plants overexpressing known positive regulators of abiotic stress tolerance, such as *ABF3*, *ABF4*, *AtXERICO*, *AtSDIR1*, *OsSDIR1*, and *GmNFYA3* [33,34,35,36,37]. The simplest explanation for this is that transgenic plants produce some compatible solutes that confer tolerance to osmotic stress but not to sodium ion toxicity.

Under salt stress, there was significant inhibition of the root growth of transgenic soybean overexpressing *lncRNA77580*. The number of lateral roots was significantly lower in transgenic soybean seedlings than in wild type (Figure S4). There was no significant difference in root development between *lncRNA77580*-OE and WT under drought stress. In saline environments, the roots of plants are the primary point of contact regarding ionic toxicity and osmotic stress. In this study, continuous salt stress from seed germination was imposed on the soybean, while for the drought stress treatment, drought stress was applied at the seedling stage, after 10 days of normal growth. Salt stress may have a more severe effect on young roots, and compared with wild type, *lncRNA77580*-OE had more DEGs under salt stress than under drought stress.

In addition to the roots, the leaves of transgenic soybean were also more severely damaged by salt stress than those of the wild type. Specifically, there was significant leaf degreening and drying in *lncRNA77580*-OE soybean under salt stress (Figure 1). Moreover, plants can generate hydrogen peroxide (H_2_O_2_) and superoxide (O^−^_2_) under salt stress. H_2_O_2_ and O^−^_2_ can be visually detected using DAB and NBT. Programmed cell death was analyzed using trypan blue. Trypan blue can color dead cells blue. However, living cells are not stained [38]. Thus, the staining results of DAB, NBT, and trypan blue indicated that the antioxidant capacity was lower in transgenic soybean than in WT. Transcriptomic profiling also indicates a greater impact of *lncRNA77580* overexpression on the expression of genes related to photosynthesis and oxidation–reduction process under salt stress. It may be that high salt stress caused more serious damage to the chloroplasts of the transgenic soybeans. High salinity has multiple effects on chloroplasts, including reduced photosynthetic efficiency, thylakoid membrane damage, oxidative stress, etc. [39]. In addition to functioning in photosynthesis, chloroplasts also contribute to the biosynthesis of amino acids, vitamins, isoprenoids, fatty acids, and lipids [40]. The dysfunction of chloroplasts caused by salt stress can have harmful effects on the physiological, biochemical, and metabolic properties of plant cells. However, many genes related to photosynthesis were upregulated in *lncRNA77580*-OE soybean under salt stress. Some genes related to oxidative stress, ion homeostasis, and energy metabolism were downregulated (Figure S5a). *lncRNA77580*-OE soybean may thus be more hypersensitive to salt stress and try to increase the energy supply under salt stress by enhancing photosynthesis, but prolonged salt stress may cause an imbalance in growth and the stress response, eventually leading to plant death [41].

Overexpression of *lncRNA77580* enhanced drought tolerance in transgenic soybean at the seedling stage and the stage from flowering to maturity (Figure 2). At the seedling stage, the leaves of WT were more likely to dry out and die than those of *lncRNA77580*-OE under drought stress. The DAB, NBT, and trypan blue staining results indicate that the antioxidant capacity was higher in transgenic soybean than in WT. Under both salt and drought stresses, the expression of genes related to oxidative stress was changed by *lncRNA77580* overexpression. However, unlike under salt stress, many genes related to oxidative stress were upregulated under drought stress (Figure S5b). Transgenic soybean may have a relatively higher ability than WT to remove excess ROS to alleviate the potential harmful effects of drought stress on soybean metabolism. Several genes differentially expressed in *lncRNA77580*-OE under salt and drought stresses were randomly selected for RT-PCR verification, and all genes exhibited the same expression tendency as shown in the RNA-seq data (Figure S6).

Furthermore, soybean plants are most sensitive to water deficit during the flowering and seed set stages, when water shortage will most seriously affect yield [42,43,44]. In this study, after 55 days in well-watered conditions, soybeans bloomed and were not watered until they were ripe. The yield (grain weight of per line) of both the WT and *lncRNA77580*-OE soybean decreased under drought, but that of the *lncRNA77580*-OE soybean was higher than that of WT under drought conditions. Drought mainly affected yield through the seed number per plant, because we found that the *lncRNA77580*-OE soybean had lower 100-seed weight and smaller seeds than wild-type soybeans (Figure S7). Water shortage during the flowering and seed set stages might influence pollen or ovule function [43], resulting in lower seed set; we found that the WT soybean had more two-seeded pods and empty pods than did the *lncRNA77580*-OE soybean under drought stress (Figure S7). Another study also reported that when water stress was imposed at the reproductive stage, there was a severe reduction in seed number, and it had different effects on different soybean varieties [43].

Genes that are genetically adjacent and co-expressed in the expression pattern are likely to be regulated by this lncRNA, which is known as the lncRNA cis mechanism of action. In this study, among the 28 genes whose expression changed due to *lncRNA77580* overexpression under different conditions, i.e., normal, salt, and drought conditions, the gene *Glyma.20G225700* is most closely positioned to *lncRNA77580* (Figure S2) and was upregulated in *lncRNA77580*-OE soybean, and similar results were found in our previous study [25]. It is predicted to be a target gene of *lncRNA77580*. The functions of a lncRNA can also be predicted by the functions of their target genes, but unfortunately, the function of *Glyma.20G225700* is unknown, and no studies have been conducted on this gene have been reported. Perhaps the molecular mechanism of *lncRNA77580* can be determined by studying the function of the gene *Glyma.20G225700*.

In conclusion, we characterized a soybean long non-coding RNA, *lncRNA77580*, through its overexpression in soybean. With the phenotypic, physiological, and molecular analyses conducted in this work, we found that overexpression of *lncRNA77580* enhances salt sensitivity while increasing the drought tolerance of transgenic plants at the seedling stage. Under water deficit at the reproductive stage, *lncRNA77580* overexpression increases the seed yield through an increased seed number per plant. *lncRNA77580* may regulate soybean response to stress by regulating a protein-coding gene. However, our comprehension of the myriad roles of *lncRNA77580* is still in its infancy. Currently, we are further exploring the functional mechanism of *lncRNA77580*.

## 4. Materials and Methods

### 4.1. Plant Transformation and Transgenic Plant Selection

For *lncRNA77580* overexpression, the full-length sequence was cloned from soybean root cDNA using the primer C3 and subcloned into the pCAMBIA3301 vector under the control of the 35S CaMV promoter.

*lncRNA77580*-overexpression constructs were electroporated into the *Agrobacterium tumefaciens* strain EHA105 and then used to transform soybean cultivar Tianlong via the *Agrobacterium*-mediated cotyledonary node transformation method. Transgenic soybean was examined using the QuickStix Kit for PAT/*bar* (EnviroLogix, America), Basta spraying, and PCR amplification of the *lncRNA77580* fragment. The expression of *lncRNA77580* in transgenic soybean was further confirmed by qPCR. The primers used in this study are listed in Table S1.

### 4.2. Evaluation of Salt and Drought Tolerance of Transgenic Plants

The WT and *lncRNA77580*-OE soybean seeds were planted in vermiculite with Hoagland nutrient solution or 100 mmol/L NaCl–Hoagland solution. The whole cultivation process was accomplished in a growth chamber with a 16 h/8 h light/dark photoperiod at 28 °C. The whole plants of 15-day-old seedlings were collected and immediately frozen in liquid nitrogen until total RNA isolation. After 35 days of continuous salt stress, the phenotype of salt stress was observed, and histochemical and physiological analyses were performed according to a previously described protocol [39]. The leaves of the WT and *lncRNA77580*-OE under normal and stress treatments for 2 days were stained with 3,3′-diaminobenzidine (DAB), nitroblue tetrazolium (NBT), and trypan blue solution (TPB) to assay H_2_O_2_, O^−^_2_, and programmed cell death. The relative water content, chlorophyll content, and electrolyte leakage were measured in the leaves of the WT and OE soybean under normal and stress treatment. Each leaf sample came from three different plants. All experiments were replicated three times.

For drought stress treatment at the seedling stage, 10 days of normal growth was followed by 5 days of drought stress treatment, and rewatering was then conducted. The growing conditions and the histochemical and physiological analyses were the same as those for the salt stress treatment mentioned above. In addition, the WT and *lncRNA77580*-OE soybean seeds were planted in the field under an auto-rain-shelter. After 55 days of growth under well-watered conditions, the soybeans bloomed and were not watered again until they were ripe. At 44 days of drought stress treatment, we observed the phenotype and measured the chlorophyll content of the WT and OE soybean leaves. After harvest, we characterized the agronomic traits of the soybean, including the grain weight per line, grain numbers per plant, and 100-grain weight.

### 4.3. RNA-Seq Analysis of Transgenic Soybean

#### 4.3.1. Library Construction and RNA Sequencing

Total RNA was extracted from the whole plant of 15-day-old WT and *lncRNA77580*-OE seedlings. The total RNA was then submitted to Personalbio in Shanghai (www.personalbio.cn) for library construction and RNA sequencing. The RNA-seq experiment was conducted with three biological replicates.

Messenger RNA was enriched by oligo(dT)-attached magnetic beads for cDNA synthesis. Size-selected and adaptor-ligated cDNA fragments were then purified for library construction. After library preparation, the libraries were sequenced on an Illumina Novaseq 6000 platform, and 150 bp paired-end reads were generated. Both generated raw data and clean data from each library were no less than 6G in sequencing depth.

#### 4.3.2. Differential Expression Analysis

All clean reads were mapped to the soybean reference genome (https://www.soybase.org/SequenceIntro.php) using HISAT2 v2.0.5. We used HTSeq (0.9.1) statistics to compare the read count values for each gene as the original expression of the gene, and FPKM was then used to standardize the expression. The difference in expression of genes was analyzed using DESeq (1.39.0) with screened conditions as follows: expression difference multiple |log_2_FoldChange| > 1, significant *p*-value < 0.05. At the same time, we used the R language Pheatmap (1.0.8) software package to perform bi-directional clustering analysis of all different genes from the samples. We obtained a heatmap according to the expression level of the same gene in different samples and the expression patterns of different genes in the same sample using the Euclidean method to calculate the distance and the Complete Linkage method to cluster. Some DEGs were verified by qPCR. The primers used this test were listed in Table S1.

#### 4.3.3. Gene Ontology Enrichment Analysis

All genes were mapped to terms in the Gene Ontology database and calculated the numbers of differentially expressed genes enriched according to each term. Using topGO (2.40.0) to perform GO enrichment analysis on the differentially expressed genes, we calculated the *p*-value using the hypergeometric distribution method (the standard of significant enrichment is *p*-value < 0.05), and we found the significantly enriched GO terms associated with differentially expressed genes to determine the main biological functions performed by these DEGs. ClusterProfiler (3.16.1) software was used to carry out the enrichment analysis of the KEGG pathways of the differentially expressed genes, focusing on the significant enrichment pathways with a *p*-value of <0.05.

### 4.4. Data Analysis

Statistical analyses were performed using Microsoft Excel, and the significance of the differences among control and treatments was analyzed by ANOVA, followed by Tukey–Kramer at a significance level of 1%.

## Data Availability

All data are available in the supplement or upon request.

## Supplementary Materials

The following supporting information can be downloaded at: https://www.mdpi.com/article/10.3390/plants12010181/s1, Figure S1: *lncRNA77580* was overexpressed in soybean; Figure S2: Common DEGs under normal condition, salt stress, and drought stress; Figure S3: Differential expression of transcription factors (TFs) in soybean seedlings with *lncRNA77580* overexpression under normal, salt, and drought conditions; Figure S4: Root number and length of WT and *lncRNA77580*-OE transgenic soybean seedlings; Figure S5: Heatmap of some DEGs in *lncRNA77580*-OE soybean; Figure S6: Quantitative RT-PCR verification for randomly selected differentially expressed genes. The data were displayed as fold changes; Figure S7: The pods and seeds of WT and OE transgenic soybean under drought stress; Table S1: Sequences of the primers used in this study.
