# Supplementary material for: Overexpression of lncRNA77580 Regulates Drought and Salinity Stress Responses in Soybean
Source: Plants (Basel). 2023 Jan 1;12(1):181. doi: 10.3390/plants12010181 (PMC9824792; doi:10.3390/plants12010181)
Supplement: Supplementary file 1 [file plants-12-00181-s001.zip › plants-2108518-supplementary.pdf]

Supplementary Material

Table S1. Sequences of the primers used in this study

Primer Name	Forward Primer(5′-3′)	Reverse Primer(5′-3′)
C3	AAGATGAAGACAAGGT	ATAGGGATAGGCAAAC
P1	TGCAGCTACATTCGGGTGAA	ATGACACGTCACAGGCAACT
Cons15	TAAAGAGCACCATGCCTATCC	TGGTTATGTGAGCAGATGCAA
Glyma.01G068200	CTCATTCTGATGCTGGTGCT	CTCGGTGTTCAACGCTTCTA
Glyma.02G136200	ATGCCCTTACCCTCACCTT	CTTGATCTGCTTTGCGTTTC
Glyma.04G220600	TGTTTGGCTCTGTTTGTCCT	GTTCCTGTTGGGGTTTGC
Glyma.06G120300	GTAAGAACTCATCACCCACCC	GTAAATCCCACTTTCCCTCC
Glyma.08G070000	CTGAGGTTAGCCAACAAGTCC	GTGGTGTAGGTGTAAGAGGGTG
Glyma.16G042700	GGGACTCAGATGAACCTTAGACT	GCAGCCACCACAACCAG
Glyma.01G203400	CAACCCATCTTTCCGTCTCA	CCAAGCATCCACCATCACTC
Glyma.18G018600	TACGAAACCACCGAACTTGT	ATCTTGTCCTTTGTAGCCCAT
Glyma.08G204800	AGGAGTGGTGCCGTGGTA	AGGGTTGTAGGGTGAAGGTG
Glyma.10G042100	CATCAGCAGCATCTGGGTT	GGTAGCGAGTGTTGGGGTC
Glyma.12G219300	GTGCTGGGCTTCCAAGTTAT	TCTAGGTGGTCCAAAAGGTTC
Glyma.12G202500	CACCCATCAAGCCCAAACC	GCTCAGAGGGACCAGCCAT
Glyma.09G073900	TTCCTCAAGCCACTGCCTCT	CGACCACCGCACCGATAA
Glyma.10G107100	GCAAAGGAATGATTCAGGG	GCAGAGTTCAACAGGAGCAA
Glyma.03G253500	CGCATCACGGCAGTAGC	ATTTGGCATTCGGTTTCAT
Glyma.12G236650	GAGCCAAAACCCAGCAATG	GGTCTCAGGAACAGGAGCAAA
Glyma.06G291700	TCCTCCCCATCAACCCAC	CCTTTTCCATCTCATTTTACCAG
Glyma.20G204500	TTGGGTTGGCTCCGTATT	AGGCTTTTGTCTTCCTTATCC

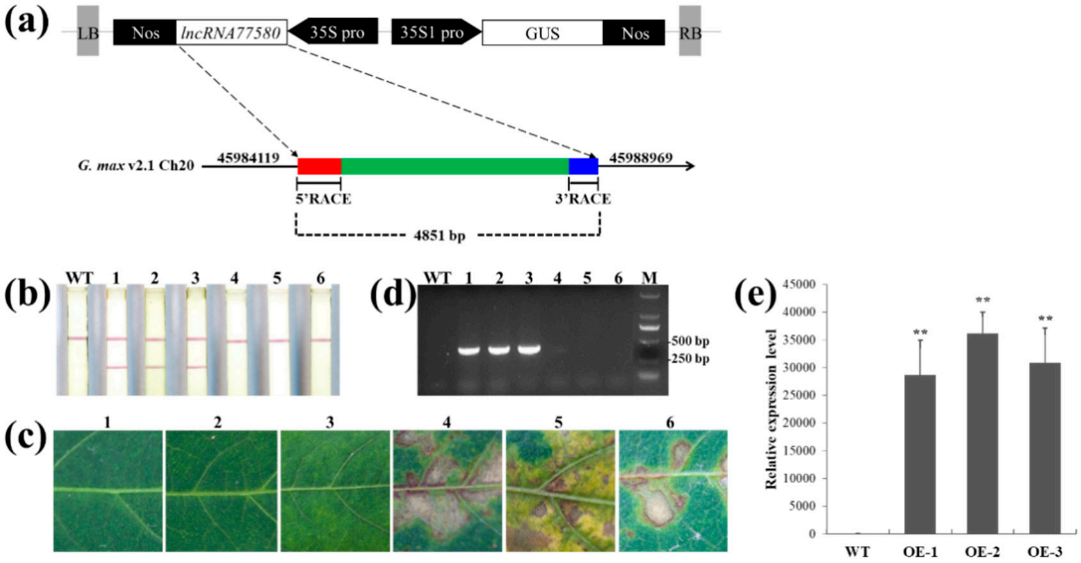

Figure S1. ***lncRNA77580*** **was overexpressed in soybean.**

(a) *lncRNA77580* was overexpressed under 35S *CaMV* promoter. Transgenic soybean was examined by using QuickStix Kit for PAT/*bar* **(b)**, Basta spraying **(c)** and PCR amplification of *lncRNA77580* fragment **(d)**. **(e)** The expression levels of *lncRNA77580* in WT and OE soybean plants. Data was shown as mean ± standard deviation (n = 3). Significant differences based on ANOVA are set at $p < 0.05$ (*) and $p < 0.01$ (**), respectively.

(a)

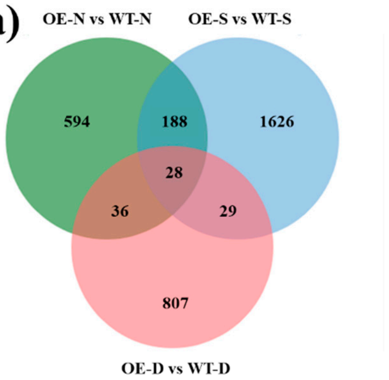

(b)

Gene ID	Regulation	Product
Glyma.20G225700	Upregulation	Protein of unknown function (DUF1685)
Glyma.17G086900	Upregulation	-
Glyma.12G236650	Upregulation	carotenoid cleavage dioxygenase 1
Glyma.12G236700	Upregulation	carotenoid cleavage dioxygenase 1
Glyma.08G270500	Upregulation	-
Glyma.11G230300	Upregulation	CRINKLY4 related 3
Glyma.07G091400	Upregulation	B-box type zinc finger protein with CCT domain
Glyma.03G231400	Upregulation	Predicted AT-hook DNA-binding family protein
Glyma.11G225500	Upregulation	UDP-Glycosyltransferase superfamily protein
Glyma.03G231500	Upregulation	Peptide chain release factor 2
Glyma.15G240600	Upregulation	senescence-related gene 1
Glyma.19G159000	Upregulation	sulfate transporter 3;1
Glyma.19G145700	Upregulation	Pectin lyase-like superfamily protein
Glyma.07G243600	Upregulation	MLP-like protein 423
Glyma.08G000800	Upregulation	ABC-2 type transporter family protein
Glyma.18G215600	Upregulation	Glycosyl hydrolase superfamily protein
Glyma.11G069500	Upregulation	laccase 11
Glyma.18G289100	Downregulation	heat shock cognate protein 70-1
Glyma.07G265000	Downregulation	Protein of unknown function (DUF506)
Glyma.01G132300	Downregulation	Protein kinase superfamily protein
Glyma.20G204500	Downregulation	-
Glyma.07G034900	Downregulation	lipoxygenase 1
Glyma.16G007500	Downregulation	proline-rich family protein
Glyma.16G030600	Downregulation	golgin candidate 4
Glyma.16G029600	Downregulation	POZ/BTB containin G-protein 1
Glyma.16G042700	Downregulation	atypical CYS HIS rich thioredoxin 4
Glyma.05G079550	Downregulation	Polynucleotidyl transferase, ribonuclease H fold protein with HRDC domain
Glyma.02G029900	Downregulation	UDP-glucosyl transferase 74B1

(c)

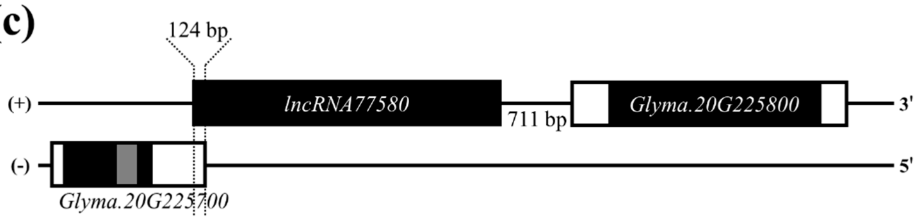

Figure S2. Common DEGs under normal condition, salt stress, and drought stress.

(a) Venn diagram of DEGs. **(b)** The 28 common DEGs in *lncRNA77580*-OE soybean under normal conditions, salt stress, and drought stress. **(c)** Location of *lncRNA77580* and its neighboring genes.

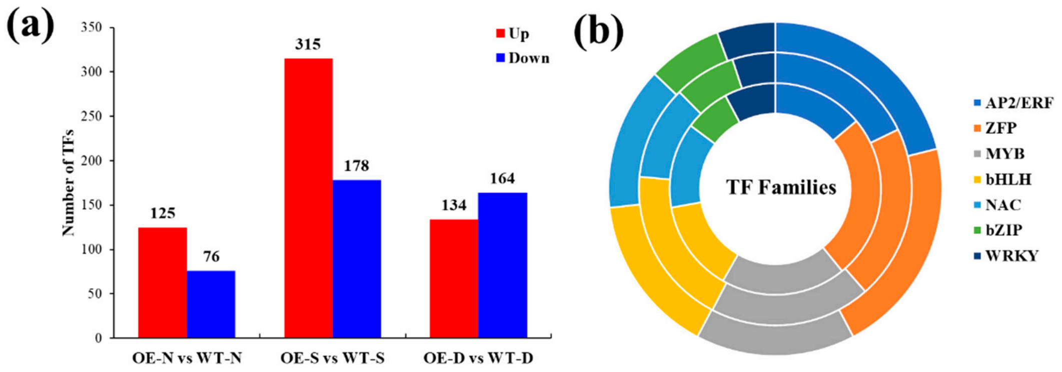

Figure S3. Differential expression of transcription factors (TFs) in soybean seedlings with *lncRNA77580* overexpression under normal, salt, and drought conditions.

(a) Numbers of up- and downregulated TFs in *lncRNA77580*-overexpression soybean. **(b)** The major TF families differentially expressed in *lncRNA77580*-OE soybean under normal (outer), salt (middle), and drought (inner) stress.

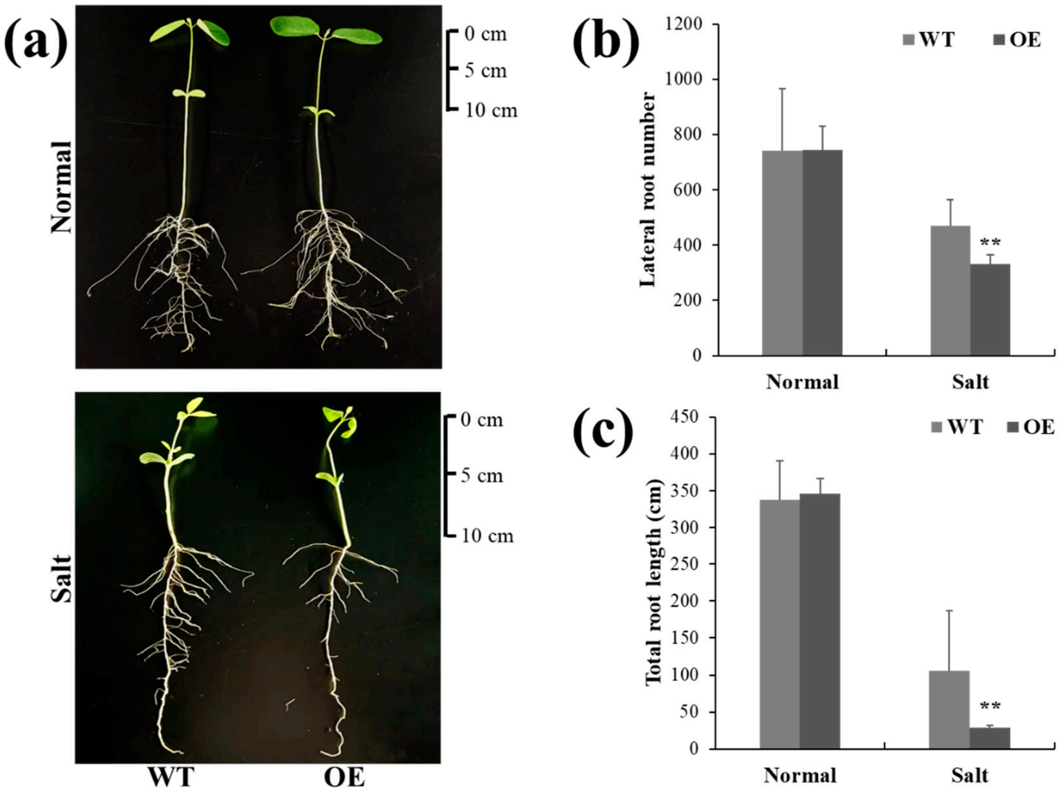

Figure S4. Root number and length of WT and *lncRNA77580*-OE transgenic soybean seedlings.

(a) Phenotypes of WT and *lncRNA77580*-OE transgenic soybean seedlings under normal conditions and salt stress for 10 days. Lateral root number **(b)** and total root length (cm) **(c)** of WT and OE transgenic soybean seedlings grown under normal conditions and salt treatment. Data are shown as the mean ± standard deviation (n = 3). Significant differences based on ANOVA were set at $p < 0.05$ (*) and $p < 0.01$ (**).

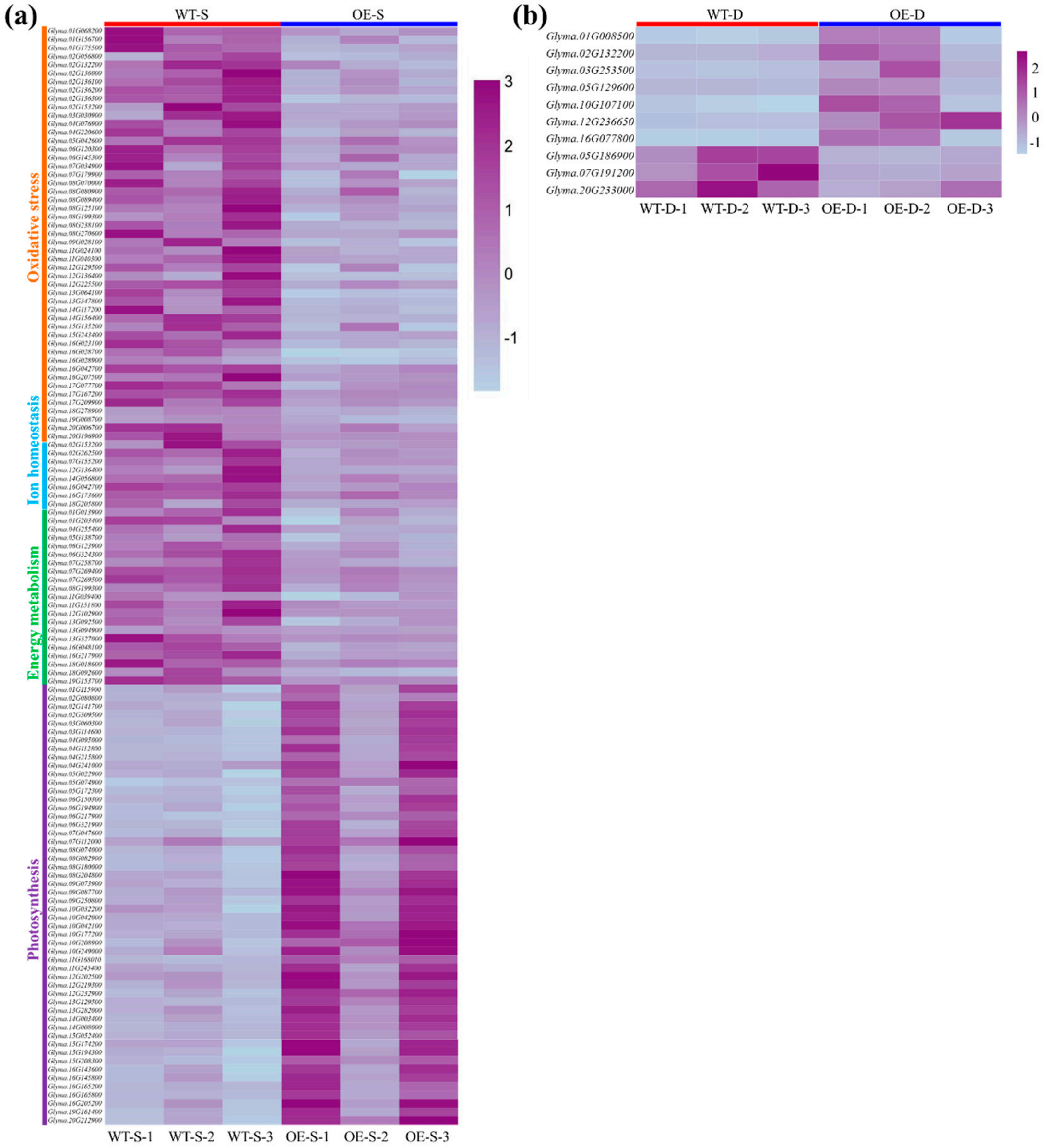

Figure S5. Heatmap of some DEGs in *lncRNA77580*-OE soybean

(a) DEGs in *lncRNA77580*-OE soybean related to oxidative stress, ion homeostasis, energy metabolism, and photosynthesis under salt stress. **(b)** DEGs related to oxidative stress in OE transgenic soybean under drought stress.

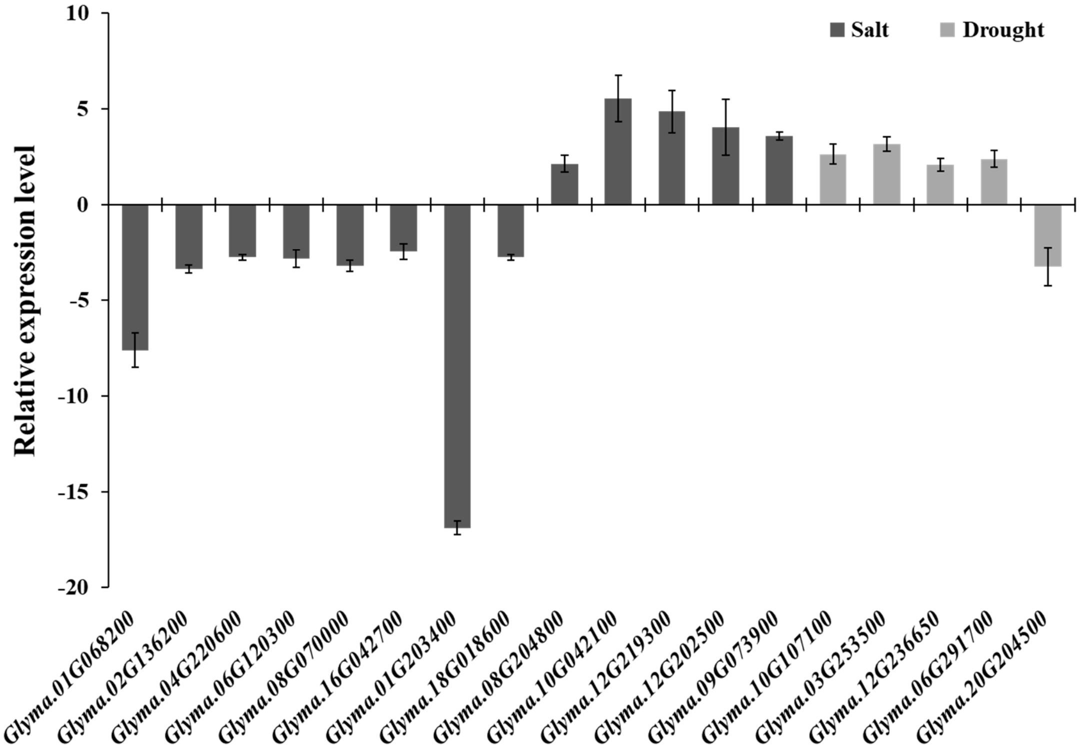

Figure S6. Quantitative RT-PCR verification for randomly selected differentially expressed genes. The data were displayed as fold changes.

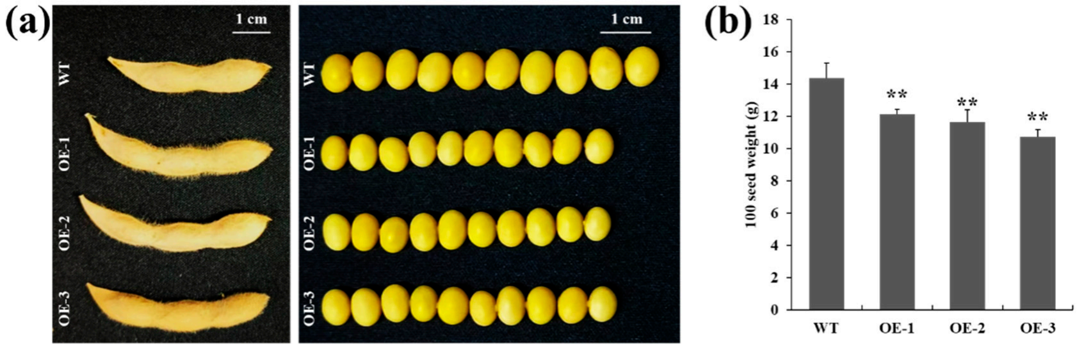

Figure S7. The pods and seeds of WT and OE transgenic soybean under drought stress.

(a) Phenotypes of pods and seeds and **(b)** 100-seed weight. Data are shown as the mean ± standard deviation (n = 3). Significant differences based on ANOVA were set at $p < 0.05$ (*) and $p < 0.01$ (**).
